# The development of a measure of participation in adults with hearing loss: a qualitative study of expert views

**DOI:** 10.1186/1745-6215-16-S1-P30

**Published:** 2015-05-29

**Authors:** Eithne Heffernan, Neil Coulson, Helen Henshaw, Johanna Barry, Melanie Ferguson

**Affiliations:** 1 NIHR Nottingham Hearing Biomedical Research Unit, Division of Clinical Neuroscience, School of Medicine, University of Nottingham, Nottingham, NG1 5DU, UK; 2 Division of Rehabilitation and Ageing, School of Medicine, University of Nottingham, Nottingham, NG7 2UH, UK; 3 MRC Institute of Hearing Research Clinical Section, Queens Medical Centre, Nottingham, NG7 2UH, UK

## Background

The International Classification of Functioning, Disability and Health (ICF) proposes that there are three primary healthcare outcomes: body functions/structure, activity and participation [1]. The ICF has recently been applied to hearing loss [2]. This is an important development, as it recognises that hearing loss can substantially affect both physical functioning and participation [3]. However, despite the importance of participation, this construct has proven difficult to measure [4]. This is primarily because participation is highly individual and because the current conceptualisation of participation is weak. The ultimate aim of this research is to develop a measure of participation in adults with hearing loss that addresses these difficulties, as presently there is no gold standard measure available [5]. The first step was to conduct a qualitative study to further develop the conceptualisation of participation in adults with hearing loss, thereby providing a foundation for its measurement.

## Method

Semi-structured interviews were conducted with nine hearing healthcare professionals and 25 adults with mild-to-moderate hearing loss. Maximum variation sampling facilitated the recruitment of participants with a variety of characteristics and experiences [6]. The data were analysed in accordance with established thematic analysis guidelines [7]. The analysis was informed by the self-regulatory model [8]. The validity of the analysis was enhanced by comparing it to an independent analysis of a sample of the interview transcripts performed by a second researcher [9].

## Results

The results show that hearing loss can lead to participation difficulties in various social domains, including work, friendship and family. Most of the interviewees with hearing loss successfully participated in some social domains, but struggled in other social domains. This was particularly problematic when they struggled in the social domains they most valued, such as family life. Some reported that, although they had many social interactions, they felt isolated during those interactions. Hearing loss can cause participation difficulties by leading to fatigue, embarrassment and diminished self-confidence in social interactions.

## Conclusions

This research demonstrates that it is important to measure individuals’ active participation in the social domains that they most value. This contrasts with many previous participation measures that assess how often people participate or the number of social domains in which people participate [10]. The next step is to review existing measures and the ICF in order to identify any additional content that should be captured in the new measure. Subsequently, the content validity and psychometric properties of the measure will be assessed.
